# An Internal Promoter Drives the Expression of a Truncated Form of *CCC1* Capable of Protecting Yeast from Iron Toxicity

**DOI:** 10.3390/microorganisms9061337

**Published:** 2021-06-20

**Authors:** Catarina Amaral, Cristina Teixeira Vicente, Soraia Marques Caetano, Ana Gaspar-Cordeiro, Yang Yang, Peter Cloetens, Célia V. Romão, Claudina Rodrigues-Pousada, Catarina Pimentel

**Affiliations:** 1 ITQB NOVA, Instituto de Tecnologia Química e Biológica António Xavier, Universidade Nova de Lisboa, Av. da República, 2780-157 Oeiras, Portugal; cristina.mt.vicente@gmail.com (C.T.V.); soraia.cmc@gmail.com (S.M.C.); carolinacordeiro@itqb.unl.pt (A.G.-C.); cmromao@itqb.unl.pt (C.V.R.); claudina@itqb.unl.pt (C.R.-P.); 2 ID16A Beamline, ESRF-The European Synchrotron, 38043 Grenoble, France; yangyang.esrf@gmail.com (Y.Y.); cloetens@esrf.fr (P.C.)

**Keywords:** yeast, iron, gene expression, alternative promoter, vacuole, transcription, toxicity, iron metabolism

## Abstract

In yeast, iron storage and detoxification depend on the Ccc1 transporter that mediates iron accumulation in vacuoles. While deletion of the *CCC1* gene renders cells unable to survive under iron overload conditions, the deletion of its previously identified regulators only partially affects survival, indicating that the mechanisms controlling iron storage and detoxification in yeast are still far from well understood. This work reveals that *CCC1* is equipped with a complex transcriptional structure comprising several regulatory regions. One of these is located inside the coding sequence of the gene and drives the expression of a short transcript encoding an N-terminally truncated protein, designated as s-Ccc1. s-Ccc1, though less efficiently than Ccc1, is able to promote metal accumulation in the vacuole, protecting cells against iron toxicity. While the expression of the s-Ccc1 appears to be repressed in the normal genomic context, our current data clearly demonstrates that it is functional and has the capacity to play a role under iron overload conditions.

## 1. Introduction

Iron (Fe) is an essential metal for almost all living organisms. However, the same chemical properties that make iron such a central element for life also make it a potential threat, as iron can generate reactive oxygen species through Fenton reactions [1].

To maintain iron intracellular concentrations within adequate levels to meet cellular needs, organisms have developed mechanisms to efficiently deal with iron fluctuations (reviewed in [2,3,4]). In iron deficiency conditions, the yeast *Saccharomyces cerevisiae* responds by triggering a complex rearrangement of gene expression mainly orchestrated by the low iron responsive transcription factor Aft1, and to a lesser extent by Aft2 (reviewed in [5]). Under such conditions, Aft1/2 accumulate in the nucleus [6,7] and activate the transcription of a set of genes, encoding proteins that promote the uptake of iron and its mobilization from internal stores, while re-directing cell metabolism towards less iron-consuming pathways [8,9,10]. In yeast, much less is known with regards to the iron excess coping mechanisms. In such conditions, the cells’ immediate response to mitigate iron toxicity effects is to restrict iron uptake by decreasing the expression of iron transporters. This approach involves several transcriptional and posttranscriptional strategies that work synergistically to prevent the intracellular accumulation of iron beyond homeostatic needs and the development of an iron overload state [11,12,13,14]. In mammalian systems, iron homeostasis is regulated at the systemic and cellular level by several mechanisms. One such mechanism involves the protein ferritin, which serves as an intracellular iron storage, releasing it in a controlled fashion. Such proteins or homologues, however, do not exist in yeast cells where the major iron detoxification pathway relies on its sequestration in the vacuole. Vacuoles are major iron reservoirs, accounting for 75% of the yeast total iron storage under normal physiological conditions [15,16]. Iron accumulation in these organelles is mainly mediated by the transporter Ccc1, encoded by the gene *CCC1*, and only marginally by the cell endocytic activity [12,17]. As such, deletion of *CCC1* renders cells unable to survive under iron overload conditions [17].

Ccc1 is ubiquitous among fungi and some species have two paralogue genes [18]. Ccc1 orthologues (also known as Vit1) exist in several other organisms [19,20,21,22,23,24,25,26] and are capable of rescuing the lethal phenotype of a knock out Ccc1 yeast strain, indicating a high degree of functional conservation throughout evolution. In plants Vit1 localizes in the vacuole where it functions as an iron importer [20,21,27] and a Vit1 crystal structure has been recently reported [22].

In *Saccharomyces cerevisiae*, the transcription factor Yap5, whose activity is regulated by two [2Fe-2S] clusters [28], was proposed to mediate iron storage through the induction of *CCC1* gene expression [29], in response to mitochondrial Fe-S biogenesis rates [30]. However, we and other authors have shown that (i) *CCC1* deletion has a very severe impact on yeast growth under Fe overload conditions, compared to *YAP5* deletion [28,31]; and (ii) deletion of the Yap5 consensus bindings sites (YREs) from the promoter region of *CCC1* does not affect iron tolerance [31], suggesting that Yap5 is not the sole regulator of vacuolar iron storage. Accordingly, the kinase Snf1 and the general stress transcription factors Msn2 and Msn4 were recently described to act synergistically with Yap5 to regulate *CCC1* transcription [32]. Nevertheless, deletion of all these regulators does not completely eliminate *CCC1* expression and thus only moderately compromises yeast resistance to excess iron [32]. Therefore, while a great deal of progress has been made in understanding the transcriptional regulation of iron storage in yeast, there are still additional mechanisms to be uncovered.

In this work we have uncovered one additional mechanism of *CCC1* regulation that leads to the expression of a functional in-frame amino-terminal truncated protein still capable of mediating iron accumulation in the vacuole. The existence of this alternative transcriptional pathway of *CCC1* reinforces the idea that Ccc1 might be regulated through several different mechanisms.

## 2. Materials and Methods

### 2.1. Yeast Strains and Growth Conditions

*Saccharomyces cerevisiae* strains (BY4742) used in this study are listed in Table S1. Mutant strains were generated using the micro homology PCR method [33]. Mutants were confirmed by PCR analysis of genomic DNA using specific primers (Table S2). Yeast strains were grown at 30 °C in synthetic complete media (SC: 0.67% ammonium sulphate-yeast nitrogen base without amino acids (Difco), 0.60% Bacto™ Casamino Acids (Difco) 2% D-glucose, supplemented with the appropriate amino acids, according to the strains’ auxotrophic markers) or SC lacking specific requirements (SD). Spot assays were carried out by spotting 5 µL of early exponential phase cultures sequentially diluted (5 × 10^3^ to 10 cells) in medium with 2% agar (Fisher BioReagents), containing the indicated concentration of FeSO_4_ (Merck).

### 2.2. Plasmids

All plasmids used in this work as well as the strategy used to generate them, are detailed in Table S3. PCR amplifications were performed using Phusion High Fidelity DNA polymerase (ThermoScientific). The sequence of all oligonucleotides is listed in Table S2. All constructs were confirmed by sequencing.

### 2.3. Immunoblotting

Strains were grown until the exponential phase, treated with the indicated FeSO_4_ concentrations and harvested at the indicated time points. Total proteins were extracted from cell cultures as described in [31]; 100 μg of total protein were resolved in a 12% SDS-PAGE and immunoblotted with the respective antibodies. Antibody anti-HA-Peroxidase high affinity rat monoclonal antibody (Roche, Mannheim, Germany) was used to detect the HA-tagged versions of Ccc1. Antibody anti-β-Galactosidase (Sigma, Darmstad, Germany) was used to detect β-Galactosidase with secondary horseradish peroxidase-bound anti-mouse IgG antibody (Santa Cruz Biotechnology, Heidelberg, Germany). Antibody anti-Pgk1 (Life Technologies, Carlsbad, USA) was used as loading control. Signals were detected using the Super Signal West Pico or West Femto Maximum Sensitivity Substrates (Thermo Fisher Scientific, Rockford, USA).

### 2.4. Protein Stability Assays

Yeast cells transformed with −690*CCC1-HA* or +*110CCC1-HA* were grown in an SD medium until the early exponential phase and left unstressed or exposed to 2 mM of FeSO_4_ for 30 min (time point 0). Cell cultures were then treated with 100 μg/mL of cycloheximide (CHX) and harvested at the indicated time points. Proteins were extracted by mechanical disruption, resolved in a 12% SDS–PAGE gel and transferred to a nitrocellulose membrane and detected as described above. Figures depicting the stability assays are representative of two independent experiments.

### 2.5. β-Galactosidase Assays

BY4742 wild type cells were transformed with the plasmids YEp356R-58 and YEp356R+1. β-Galactosidase activity was monitored in solid media as described previously [34]. Cultures were grown in SD liquid medium until the stationary phase and 3.5 × 10^7^ cells were spotted. After 90 min incubation at 30 °C, cells were overlaid with X-gal buffer (*lacZ* buffer pH 7, 60 mM Na_2_HPO_4_, 40 mM NaH_2_PO_4_, 10 mM KCl, 1 mM MgSO_4_, 0.5% (*w*/*v*) agarose (NZYTech), 2% (*w*/*v*) X-gal (NZYTech)) and incubated at 37 °C until the development of the blue color.

### 2.6. Fluorescence Microscopy

The BY4742 strain, carrying a mCherry tagged genomic version of *ZRC1* (*ZRC1-mCherry*), was transformed with the plasmids GFP-*s-CCC1* and grown until the exponential phase. The DNA dye 4’,6-diamidino-2-phenylindole (DAPI) (Sigma-Aldrich, Darmstad, Germany) was added directly to the culture 10 min before collection at a final concentration of 25 μg/mL. After washing with phosphate-buffered saline (PBS), cells were re-suspended. Confocal images were acquired on a Zeiss LSM 880 point scanning confocal microscope using the Airyscan detector, a 63× Plan-Apochromat 1.4NA DIC oil immersion objective (Zeiss) and the 405 nm, 488 nm and 561 nm laser lines. The Zeiss Zen 2.3 (black edition) software was used to control the microscope, adjust spectral detection for the emission of DAPI, GFP and mCherry and for processing of the Airyscan raw images. The figure shown is representative of at least two independent experiments.

### 2.7. Synchrotron X-ray Fluorescence Imaging (SXRF)

Cells were grown until the exponential phase, treated or left untreated with 250 μM FeSO_4_ for 30 min, washed with and re-suspended in ultra pure water; 10 μL of the cell suspension were spotted in Silicon Nitride Membranes 1.5 mm × 1.5 mm, 500 nm thickness (Silson Ltd, England) and manually blotted. Membranes were plunge frozen in liquid ethane using a Leica EMGP. Frozen samples were transferred to a cryo-box in a Leica VCM and kept in liquid nitrogen until analysis. SXRF experiments were performed under vacuum on a cryo sample stage, at the Nano-Imaging beamline ID16A-NI of the European Synchrotron Radiation Facility (ESRF, Grenoble, France). A pair of multilayer coated fixed curvature Kirkpatrick-Baez (KB) focusing mirrors provides a hard X-ray nanoprobe at 17 keV (~30 nm) at very high flux of 4.1 × 10^11^ ph/s within a broad bandpass (1%) [35]. The sample stayed in the focal plane of the nanoprobe, being fly scanned with a sampling interval of 15 nm and a transit time of 50 ms. Consequently, XRF signal from all biological-relevant elements (K, P, Zn, Fe, etc.) can be detected by collecting fluorescence emission photons with a 6-element silicon drift detector (Rayspec, England). The detector sits orthogonal to the incoming beam. The SXRF signal, so called fluorescence spectra, was fitted by PyMCA software [36] to calculate the elemental distribution maps for the detected elements [35]. At least 3 images were recorded per condition and strain, a single-cell representative image per condition and strain is shown.

### 2.8. Northern Blot

Total RNA was isolated from exponentially growing cells using the RNeasy kit (Qiagen) following the manufacturer’s instructions.

For Northern blotting approximately 40 μg of RNA were loaded in a formaldehyde gel and transferred to a nylon membrane, (GE Healthcare, Freiburg, Germany). Specific ribo-probe was obtained by in vitro transcription using ^32^P-UTP (PerkinElmer) and T7 RNA polymerase (Promega, Madison, USA). The transcription template consisted of a fragment of *CCC1* amplified by PCR with the primers T7_*CCC1*_Rv and *CCC1*_2ATG_Fw (Table S2). For the wild type strain a specific DNA probe was obtained by in vitro transcription using ^32^P-ATP (PerkinElmer) and MegaPrime kit (GE Healthcare) following the manufacture instructions. The template was amplified by PCR with the primers *CCC1*-F and *CCC1*-R (Table S2). Radiolabeled probes were purified using G25 Microspin columns (GE Healthcare,Freiburg, Germany). Membranes were hybridized overnight with radiolabeled specific probe in PerfectHyb Plus Hybridization Buffer (Sigma Aldrich) at 68 °C.

### 2.9. Real-Time RT-PCR Analyses (qRT-PCR)

Cells were grown until early the exponential phase and left untreated or treated with 2 mM of FeSO_4_. Cultures were harvested at the indicated time points and RNA was isolated using RNeasy kit (Qiagen). RNA samples were next treated with DNase - TURBO™ DNase-free (Ambion, Vilnius, Lithuania), according to the manufacturer’s instructions and purified. Total RNA (1 μg) was reverse transcribed with Transcriptor Reverse Transcriptase (Roche Diagnostics). qPCR reactions were performed in the Light Cycler 480 II Real-Time PCR System (Roche), using Light Cycler 480 SYBR Green I Master (Roche, Mannheim, Germany). Relative standard curves were draw for each gene, using triplicate serial dilutions of cDNA. Relative expression was determined by the relative quantification method with efficiency correction, using the LightCycler 480 Software 1.5 (Roche). The expression of the actin gene (*ACT1*) was used as a reference. All assays were made using biological and technical triplicates. 

## 3. Results

### 3.1. A Promoter Region of 58 bp Maintains the Expression of CCC1 at Levels That Allow Cells to Overcome Iron Toxicity

**Figure 1:**
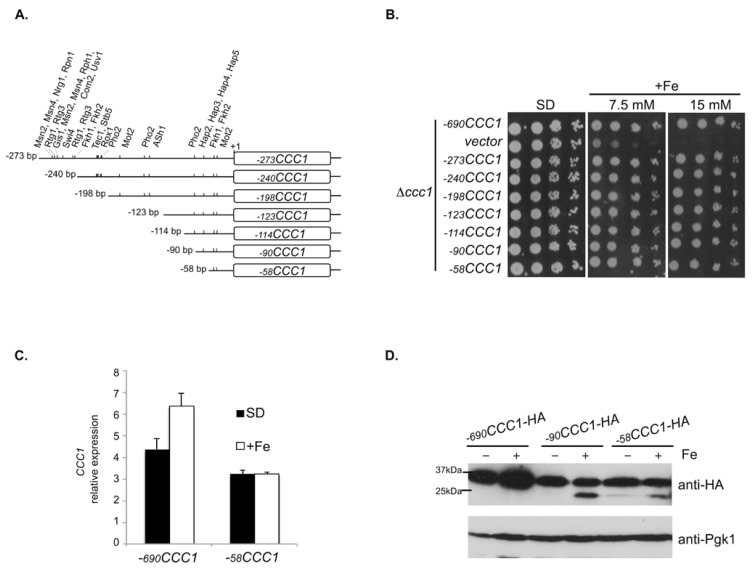
Functional analysis of *CCC1* promoter. (**A**) Schematic representation of the 5′ sequential deletions of *CCC1*. Putative transcription factor binding sites are represented by vertical lines (detailed in Table S4); (**B**) 5′ deletions of *CCC1* were cloned into pRS416 (vector) and the resultant plasmids were used to transform Δ*ccc1* cells. Growth was monitored in plates supplemented (+Fe) or not (SD) with FeSO_4_. (**C**,**D**) Δ*ccc1* cells transformed with the indicated plasmids were grown exponentially in SD medium and left untreated (SD or −) or treated (+Fe) with 2 mM of FeSO_4_ for 30 min. *CCC1* expression was assessed by (**C**) qRT-PCR and (**D**) immunoblotting with an anti-HA antibody. Pgk1 was used as a loading control in the immunoblot assays.

In a previous work, we showed that the regulation of *CCC1* expression by Yap5 is not essential for yeast cells to deal with Fe loading conditions [31]. Accordingly, it was later shown that the kinase Snf1 regulates this gene via the transcription factors Msn2 and Msn4 [32]. However, deletion of all the identified regulators did not completely impair *CCC1* expression [31,32], clearly indicating that other transcriptional player(s) remain to be uncovered. To address this, we generated several constructs containing sequential 5′ deletions of the promoter sequence of the gene (Figure 1A). The expression of *CCC1* driven by the construct −*273CCC1* is independent of Yap5 [31], as the functional YRE is absent, and therefore sequential deletions were made downstream of this region, ranging from −273 to −58 bp upstream of the AUG initiation codon (Figure 1A). Deletions took into account the presence of consensus transcription factor binding sequences, found in the 273 bp region upstream of the AUG, according to the YEASTRACT database [37] (Figure 1A and Table S4). The constructs were used to transform *CCC1* knock out (Δ*ccc1*) mutant cells and growth was assayed in plates containing medium supplemented with different Fe concentrations. The −*690CCC1* construct mimics the expression of the genomic copy of *CCC1* [31]. We found that cell growth under Fe overload conditions was unaffected by the promoter length (Figure 1B). Accordingly, although at lower levels, we could detect *CCC1* mRNA expression in cells carrying the shortest construct, comprising only 58 bp upstream of the translation initiation codon (−*58CCC1*, Figure 1C,D).

**Figure 2:**
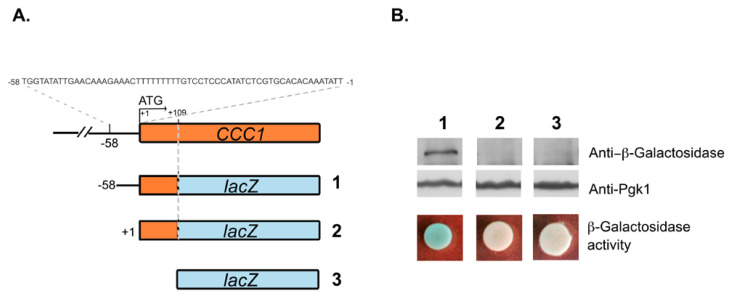
The 58 bp region located immediately upstream the translation initiation codon of *CCC1* has promoter activity. (**A**) Schematic representation of the plasmids 1 (yEp356R−58), 2 (yEp356R+1) and 3 (vector yEp356R). In 1, the 58 bp region of *CCC1* promoter along with the first 109 bp of the coding sequence of *CCC1* were cloned in frame with *lacZ*. In 2, only the 109 bp region was cloned in frame with the reporter gene sequence. (**B**) Wild type cells were transformed with plasmids 1, 2 or 3 and β-galactosidase levels were detected by immunoblotting, using an anti-β-galactosidase antibody. Pgk1 levels were used as a loading control. β-galactosidase activity was assayed by spotting 3.5 × 10^7^ stationary phase cells transformed with the indicated constructs in SD plates. Plates were incubated at 30 °C, until the development of blue color, after overlay with X-gal/agarose mixture.

To determine whether the 58 bp region has bona fide promoter activity, we cloned a fragment from −58 to +109 bp relative to the initiation codon in front of a promoter-less *lacZ* gene (Figure 2A). The resultant plasmid was used to transform wild type (WT) cells and the reporter expression was assessed by immunoblotting using an anti-β-galactosidase antibody and by in vivo plate assay using X-gal/agarose overlay. As a control, the sequence encompassing the region from +1 to +109 bp was also cloned upstream of the *lacZ* gene (Figure 2A). Results from this experiment show that the 58 bp fragment was able to drive the expression of the reporter gene, providing compelling evidence that this small region has promoter activity (Figure 2B).

### 3.2. Deletions Extending into the CCC1 Coding Sequence Lead to the Expression of a Functional In-Frame N-Terminal Truncated Form of Ccc1

**Figure 3:**
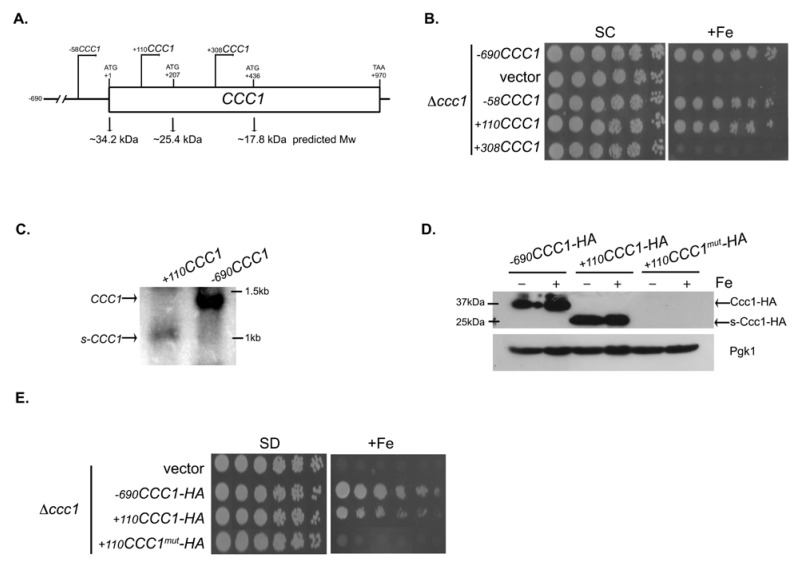
Deletions of *CCC1*, extending into the open reading frame, rescue the growth defect of the mutant Δ*ccc1* due to the expression of an alternative, in frame, truncated version of the gene. (**A**) Schematic representation of the *CCC1* gene. In frame ATGs and the molecular weight of the corresponding putative, in frame, truncated isoforms are showed. Angular arrows denote gene truncations carried by the indicated constructs. (**B**) Exponentially growing Δ*ccc1* cells transformed with the indicated plasmids or with pRS416 (vector), were serially diluted, spotted onto SD plates supplemented or not with 10 mM of FeSO_4_ and grown for 2 days at 30 °C. (**C**) Exponentially growing Δ*ccc1* cells expressing *s-CCC1* (+*110CCC1*) or *CCC1* (−*690CCC1*) were harvested and total RNA was analyzed by Northern blotting with a *CCC1* ribo-probe. (**D**) Protein extracts from Δ*ccc1* cultures carrying the plasmids expressing the indicated HA tagged *CCC1* versions and left untreated (−) or treated (+) with FeSO_4_ for 30 min, were analyzed by immunoblotting using an anti-HA antibody. Pgk1 levels served as loading control. Mutation of the triplet ATG located at position +207 (+*110CCC1*^mut^-HA) eliminates expression of the truncated isoform and (**E**) renders Δ*ccc1* unable to grow in medium supplemented with 7 mM of FeSO_4_ (+Fe).

A careful inspection of our immunoblot assays revealed the recurring appearance of a 25 kDa cross-reactive lower molecular weight band in Δ*ccc1* cells carrying the constructs −90*CCC1-HA* and −*58CCC1-HA* (Figure 1D), but not longer constructs (Figure S1). Thus, we hypothesized that yeast cells may express an in-frame N-terminal truncated form of the protein. To test this, we extended the 5′ deletions of the *CCC1* gene further 3′ and generated two plasmids without a portion of the gene coding sequence (+110*CCC1* and +308*CCC1*). Deletions were made taking into account the in-frame internal methionine codons predicted to act as putative start codons, found at positions +207 bp and +436 bp (Figure 3A). While the construct +110*CCC1* was capable of rescuing the growth defect of the Δ*ccc1* mutant, the construct +308*CCC1* was not (Figure 3B). Identical results were obtained when we used a different vector or integrated such gene fragments into the genome of the Δ*ccc1* strain, ruling out the possibility of a technical artifact (Figure S2).

To confirm the presence of an in-frame N-terminal truncated form of Ccc1 in Δ*ccc1* cells transformed with the +110*CCC1* construct, we analyzed the size of *CCC1* transcripts and the molecular weight of a C-terminus HA-tagged version of the protein by Northern and Western blotting, respectively. As expected, removal of the first 110 bp from the gene coding region triggers the expression of a shortened version of Ccc1, hereinafter designated as s-Ccc1, detected at both the RNA (Figure 3C) and protein (Figure 3D) levels. 

The molecular weight of s-Ccc1 (approximately 25 kDa) suggests that translation initiates at the AUG codon located at position +207 bp (Figure 3A). We therefore mutated the corresponding methionine to leucine (M70L) in the +110*CCC1-HA* construct and introduced the resulting plasmid, +110*CCC1-HA^mut^*, into Δ*ccc1* cells. Results from this experiment show that the M70L mutation completely abrogates protein expression (Figure 3D), confirming that the +207 AUG is the initiation codon of the truncated protein. Accordingly, cells containing this plasmid were no longer able to support excessive Fe concentrations (Figure 3E).

Together, our findings sustain the existence of a secondary promoter within the coding region of *CCC1*, which drives the expression of an in-frame functional N-terminal truncated proteoform.

### 3.3. The Genomic Context Controls s-CCC1 Expression

**Figure 4:**
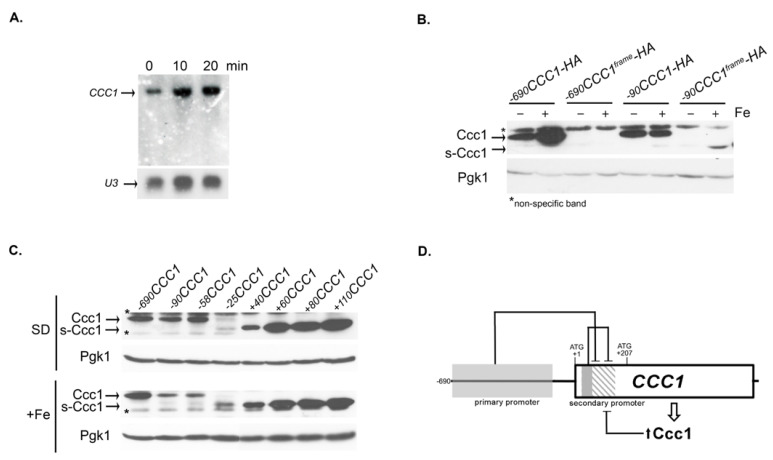
*s-CCC1* expression is repressed in its natural genomic context. (**A**) Exponentially growing cells (wild type) were exposed to 2 mM FeSO_4_ and *CCC1* gene expression was analyzed by Northern blot, at the indicated time points, *U3* was used as a loading control. (**B**,**C**) Δ*ccc1* cells carrying the indicated constructs were grown to mid-exponential phase and left untreated (-SD) or treated (+) with 2 mM FeSO4, for 30 min. Protein extracts were analyzed by immunoblotting using anti-HA antibody. Pgk1 levels were used as a loading control. Asterisks indicate non-specific bands. (**D**) Schematic representation of *s-CCC1* repression.

While the *s-CCC1* isoform is abundantly expressed in Δ*ccc1* cells carrying the +*110CCC1* plasmid, we were unable to detect it by immunoblotting in cells transformed with the full-length promoter plasmid (−*690CCC1-HA*, Figure 1D and Figure 3D), or by Northern blotting in wild-type cells (Figure 4A). These findings raised the hypothesis that elements upstream of +110 bp might be inhibiting *s-CCC1* expression. Corroborating this assumption was the previous observation that cells transformed with plasmids missing a great part of the *CCC1* promoter region (plasmids −*90CCC1-HA* and −*58CCC1-HA*) express *s-CCC1*, albeit at low amounts (Figure 1D). To investigate if the emergence of s-Ccc1 in such genomic contexts was a consequence of the reduction of Ccc1 protein levels or resulted from the elimination of repressive elements, we disrupted the open reading frame (ORF) of *CCC1* by inserting an extra nucleotide at position +6 of the coding region in the −*690CCC1-HA* and −90*CCC1*-*HA*, thereby generating the plasmids −*690CCC1^frame^-HA* and −90*CCC1^frame^-HA*, respectively. Immunoblot analysis of cells transformed with these constructs confirmed the successful elimination of Ccc1 expression and revealed that, while the ORF disruption did not trigger s-*CCC1* expression in cells transformed with the plasmid −*690CCC1^frame^-HA*, it increases s-*CCC1* levels in cells carrying the plasmid −90*CCC1^frame^-HA* (Figure 4B). Accordingly, the double deletion of *CCC1* known regulators, Yap5 and Snf1 [32] drastically reduced *CCC1* expression, however, did not induce s-Ccc1 appearance (Figure S3). These findings suggest that elements located in the primary promoter, positively regulate the expression of the longer Ccc1 isoform, but hinder the activity of the internal secondary promoter. This hypothesis is further supported by deletion analysis of the primary promoter (Figure 1D and Figure S1). It appears that in the absence of such inhibition (construct −90*CCC-HA*) the elimination of Ccc1 (−90*CCC1^frame^-HA*) increases the levels of the shortened proteoform, being more evident under Fe overload conditions.

The lower levels of s-Ccc1 observed in strains carrying plasmids −*90CCC1-HA* and −*58CCC1-HA* (Figure 1D) when compared with cells transformed with the plasmid +*110CCC1* (Figure 3D) indicate the presence of other downstream modulators of *s-CCC1* repression. Therefore, we inspected the region between −58 and +110 bp through deletion analysis and followed the expression of s-Ccc1-HA by immunoblotting, under Fe replete and overload conditions (Figure 4C). We found that the region located between −58 and −25 bp is critical for the expression of the longer isoform. No variation in s-Ccc1 levels was observed in deletions extending downstream +60 bp (Figure 4C). The higher levels of s-Ccc1 in cells expressing +*60CCC1-HA* compared to those expressing +*40CCC1-HA* suggest a strong negative determinant of s-*CCC1* expression located in the region between +40 and +60 bp (Figure 4C).

We also tried to find a specific stress condition that could derepress the expression of *s-CCC1*. We tested, by immunoblotting, Ccc1 expression driven by the construct −*690CCC1-HA*, after treatment with MnCl_2_, CaCl_2_, CuSO_4_, CoSO_4_, several carbon sources (raffinose, galactose, glycerol and ethanol), prolonged treatment with excess of iron (250 min), hypoxia, anoxia, growth for four days and bathophenanthroline disulfonate (BPS). None of the tested conditions, however, triggered the appearance of s-Ccc1 (data not shown).

Together, our results indicate that *s-CCC1* expression is strongly repressed in its normal genomic context. The repression is exerted at least at three different levels, namely by (i) the *CCC1* primary promoter, (ii) elements located within *CCC1* coding sequence and (iii) Ccc1 protein levels (Figure 4D).

### 3.4. s-Ccc1 Locates in the Endoplasmic Reticulum and Vacuole and Is More Stable but Less Efficient Than Ccc1 in Iron Detoxification

By focusing on protein stability, Fe responsiveness, subcellular localization and activity, we investigated whether the truncated protein may exhibit distinct properties that could constitute an advantage (or disadvantage) with regards to Fe toxicity protection.

**Figure 5:**
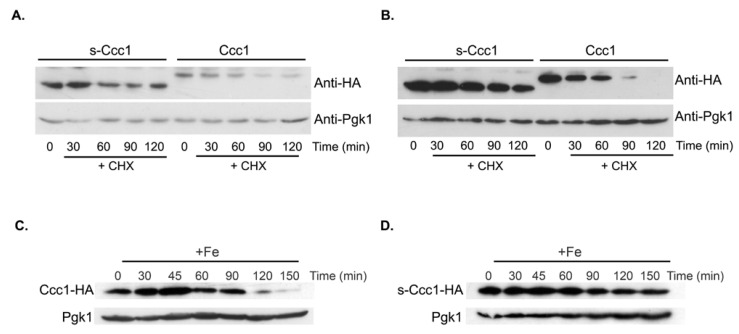
s-Ccc1 is more stable than Ccc1. Ccc1-HA and s-Ccc1-HA protein stability was assessed by cycloheximide (CHX) chase assay. Δccc1 expressing either the Ccc1 or s-Ccc1 (plasmids −690CCC1-HA and +110CCC1-HA, respectively) were grown to mid-exponential phase and left untreated (**A**) or treated (**B**) with 2 mM of FeSO_4_ for 30 min. CHX was then added to a final concentration of 100 μg/mL. Cells were collected at the indicated time points and protein extracts were immunobloted using an anti-HA antibody. Exponentially growing Δccc1 cells, expressing (**C**) Ccc1-HA or (**D**) s-Ccc1-HA, were treated with 2 mM FeSO_4_, harvested at the indicated time points and examined by immunoblotting using an anti-HA antibody. Pgk1 levels were used as a loading control.

As such, Ccc1-HA and s-Ccc1-HA protein stability was evaluated by cycloheximide (CHX) chase assays. Briefly, Δ*ccc1* cells were transformed with −690*CCC1-HA* or +*110CCC1-HA* plasmids to monitor Ccc1-HA and s-Ccc1-HA expression, respectively. Cells were left untreated or treated with 2 mM of FeSO_4_ for 30 min, exposed to 100 µg/mL of CHX and harvested at the indicated time points (Figure 5). We found that the presence of Fe in the medium increases the degradation rate of both proteoforms, with s-Ccc1 being always more stable than Ccc1 (Figure 5A,B and Figure S4). Interestingly, the RAPID prediction tool [38], revealed that Ccc1 has a higher percentage of intrinsically disordered residues (61 out of 322 residues; 18.9%) when compared to s-Ccc1 (3 out of 253 residues; 1.2%), which is a known determinant of protein degradation rates. Moreover, while Ccc1 levels respond readily to Fe availability, peaking at 45 min after treatment and then rapidly decreasing, the levels of s-Ccc1 do not decrease as rapidly over the treatment period (Figure 5C,D).

**Figure 6:**
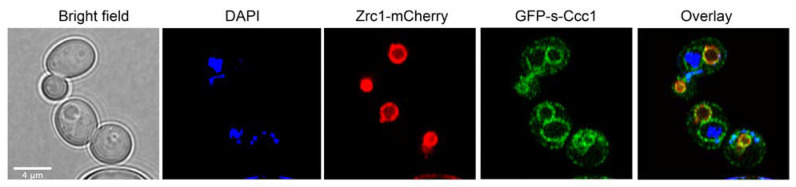
s-Ccc1 is located on ER and vacuole membranes. A strain with a genomic mCherry tagged copy of *ZRC1* was transformed with a plasmid encoding the fusion GFP-s-Ccc1. Cells were grown to mid-exponential phase and collected. DAPI was used for nuclei staining. Live cells were imaged by confocal microscopy under control conditions (SD).

Next, we fused the N-terminal region of s-Ccc1 to GFP and assessed the localization of the resultant chimera by confocal microscopy. To facilitate the identification of cell vacuoles, we used a strain that expresses the vacuolar zinc transporter Zrc1, fused to mCherry at the C-terminus. Results from this experiment showed that, while Ccc1 localizes in the vacuole (Figure S5), as previously described by others and [17], GFP-s-Ccc1 was found around the nucleus, a pattern consistent with endoplasmic reticulum (ER) localization, and in the vacuolar membrane (Figure 6), independently of Fe concentration in the growth medium (data not shown).

**Figure 7:**
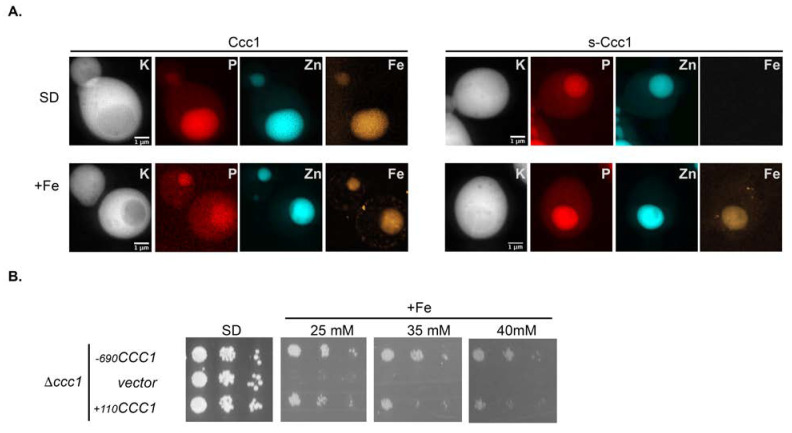
s-Ccc1 is not as efficient as Ccc1. (**A**) Synchrotron X-ray fluorescence nano-imaging distribution maps of potassium (K), phosphorus (P), zinc (Zn) and iron (Fe), in yeast cells expressing Ccc1 or s-Ccc1, under Fe replete (SD) and overload conditions (+Fe). (**B**) Exponentially growing Δ*ccc1* cells transformed with the indicated plasmids or with pRS416 (vector), were serially diluted, spotted onto SD plates supplemented or not with FeSO_4_ and grown for 2 days at 30 °C.

The unexpected ER localization of s-Ccc1 puts forward the hypothesis that the ER could be a potential Fe storage/trafficking compartment. Therefore, to monitor putative non-vacuolar Fe accumulation, we used synchrotron X-ray fluorescence imaging (SXRF), to track Fe distribution in relation to the vacuole position. Briefly, Δ*ccc1* cells expressing *CCC1* or *s-CCC1* (transformed with the plasmids −*690CCC1* or +*110CCC1*, respectively) were grown under Fe replete or overload conditions, cryo-fixed and analysed by SXRF (Figure 7A). As expected, cells expressing Ccc1 accumulate iron in the vacuole under both Fe conditions, as corroborated by co-localization with zinc and phosphorus, which are known to be stored in this compartment [39,40]. *s-CCC1* expressing cells however, only accumulate Fe in the vacuole after medium supplementation (Figure 7A), suggesting that the short isoform does not mediate the accumulation of Fe in the vacuoles as efficiently as Ccc1. Reinforcing this hypothesis, we also showed that cells that express s-*CCC1* do not grow as well as those that express *CCC1*, in extremely high concentrations of Fe (≥25 mM FeSO_4_, Figure 7B).

## 4. Discussion

The present study was aimed at exploring the poorly defined transcriptional modulators that control *CCC1* expression. Remarkably, we observed that the promoter of the gene along with part of the coding sequence could be deleted, without appreciable loss of tolerance to iron excess.

A portion of the *CCC1* gene, comprising only 58 bp upstream of the annotated AUG codon, may function as a promoter region and drive the expression of the full-length protein. This region may be of great interest from a fungal synthetic biology perspective, as minimally sized promoters are sought for advancing the field [41].

We also identified an additional promoter region within the *CCC1* coding region, which drives the transcription of a 5′ truncated mRNA resulting in an in-frame functional N-terminal truncated proteoform, that we designated as s-Ccc1. The expression of *s-CCC1* appears to be strongly dependent on the genetic context, as it is not detected in the wild type strain or in a Δ*ccc1* strain ectopically expressing the full-length protein, independently of the Fe concentrations used (Figure 1D and Figure 4A). Deletion analysis targeting the genomic region upstream of the second translation initiation codon revealed that *s-CCC1* expression is strongly repressed (Figure 4C and Figure S1). Interestingly, our data suggests that promoter deletions that strongly compromise the expression of *CCC1* are accompanied by the appearance of s-*CCC1*. In these particular genetic contexts abrogation of Ccc1 synthesis further increases s-Ccc1 expression (Figure 4B), and thus repression may as well be inversely correlated with the non-vacuolar Fe status. Moreover, the observation that Fe induces the expression of s-Ccc1 in such contexts (Figure 1D and Figure 4B) corroborates this hypothesis.

Using N-terminal proteomics approaches, several groups have reported that translation start at an in-frame downstream initiation codon is a commonly observed trait in yeast [42,43,44]. While the biological role of many of these proteoforms remains elusive, several authors have showed that selection of appropriate transcription and translation start sites might control the subcellular localization of the resulting isoforms [45,46,47,48,49,50]. Our study shows that Ccc1 localizes in the vacuolar membrane, whereas s-Ccc1 localizes both in the vacuolar and ER membranes (Figure 6). Polytopic membrane proteins such as Ccc1, are folded properly in the ER (reviewed in [51]). Our observation that s-Ccc1 accumulates in the ER is unlikely to be due to an error in folding, as some of the proteins are able reach the vacuole membrane (Figure 6) however, the exit from the ER may be compromised.

The dual localization of s-Ccc1, can potentially justify its lower efficacy in storing Fe in the vacuole (Figure 7). Alternatively, the N-terminal flexible region present in Ccc1 but not in s-Ccc1 may assist this process, since protein termini can allosterically affect transport activity (reviewed in [51]).

Corroborating the observation that alternative truncated isoforms can lead to different protein stability [52,53], we found that Ccc1 is not as stable as s-Ccc1. Fe decreases the stability of both proteoforms; being the sharpest decrease observed for Ccc1 (Figure 5). Ccc1 has a higher percentage of intrinsically disordered residues when compared to s-Ccc1, which may account for its lower stability, as unstable proteins tend to have more disordered regions [53]. Recently, Sorribes-Dauden et al. suggested that cysteine, proline and serine residues present in the N-terminus of Ccc1 may be the target of post-transcriptional modifications and therefore may be important to regulate protein stability [54].

Despite our findings, the question remains as to why *CCC1* gene has two promoters, why is the internal (secondary) promoter being repressed and what role it serves. In our view, the lower functional efficacy of s-Ccc1, along with its lower responsiveness to Fe, may justify the repression of the internal promoter in a normal genomic context. Being single-celled organisms, yeasts need to respond rapidly to Fe availability and the key players in this process need to be tuned to the rhythm of Fe fluctuations, which would otherwise endanger cellular homeostasis. Here we have shown that Ccc1 is the isoform that better serves this purpose. As such, alternative promoters may function as fail-safe mechanisms that would come into play in a scenario where *CCC1* canonical regulators fail to control gene expression, thereby ensuring cell survival under Fe overload conditions.

## Supplementary Materials

The following are available online at https://www.mdpi.com/article/10.3390/microorganisms9061337/s1: Figure S1. s-Ccc1 is repressed by elements present upstream −90 bp. Figure S2. Removal of *CCC1* promoter and part of the gene coding sequence, still rescues Δ*ccc1* high iron–associated phenotype, independently of the vector used for cloning. Figure S3. Deletion of *YAP5* and *SNF1* does not induce s-Ccc1 expression. Figure S4. s-Ccc1 and Ccc1 stability. Figure S5. Ccc1 is located in the vacuole membranes. Table S1. *Saccharomyces cerevisiae* strains used in this study. Table S2. Oligonucleotides used in the study. Table S3. Plasmids used and generated in this study. Table S4. Putative transcription factors binding sites located in the *CCC1* gene region spanning 273 bp upstream the AUG codon, according to YEASTRACT.
