# Supplementary material for: An Internal Promoter Drives the Expression of a Truncated Form of CCC1 Capable of Protecting Yeast from Iron Toxicity
Source: Microorganisms. 2021 Jun 20;9(6):1337. doi: 10.3390/microorganisms9061337 (PMC8235630; doi:10.3390/microorganisms9061337)
Supplement: Supplementary file 1 [file microorganisms-09-01337-s001.zip › microorganisms-1230148-supplementary.pdf]

An internal promoter drives the expression of a truncated form of *CCC1* capable of protecting yeast from iron toxicity

Catarina Amaral, Cristina Teixeira Vicente, Soraia Marques Caetano, Ana Gaspar-Cordeiro, Yang Yang, Peter Cloetens, Célia V. Romão, Claudina Rodrigues-Pousada, Catarina Pimentel*

Supplementary material

Figure S1. s-Ccc1 is repressed by elements present upstream -90 bp.

Figure S2. Removal of *CCC1* promoter and part of the gene coding sequence, still rescues Δ*ccc1* high iron–associated phenotype, independently of the vector used for cloning.

Figure S3. Deletion of *YAP5* and *SNF1* does not induce s-Ccc1 expression.

Figure S4. s-Ccc1 and Ccc1 stability.

Figure S5. Ccc1 is located in the vacuole membranes.

Table S1. *Saccharomyces cerevisiae* strains used in this study

Table S2. Oligonucleotides used in the study.

Table S3. Plasmids used and generated in this study.

Table S4. Putative transcription factors binding sites located in the *CCC1* gene region spanning 273 bp upstream the AUG codon, according to YEASTRACT

References

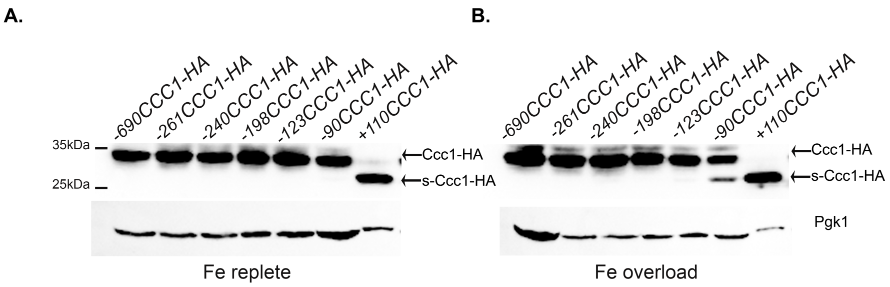

Figure S1. s-Ccc1 is repressed by elements present upstream -90 bp. *Δccc1* strain expressing the indicated constructs were grown to mid-exponential phase in the absence (A) or presence (B) of iron supplementation (2mM $FeSO_4$) and harvested. Total protein extracts were analyzed by immunoblotting using anti-HA antibody. Pgk1 was used as loading control.

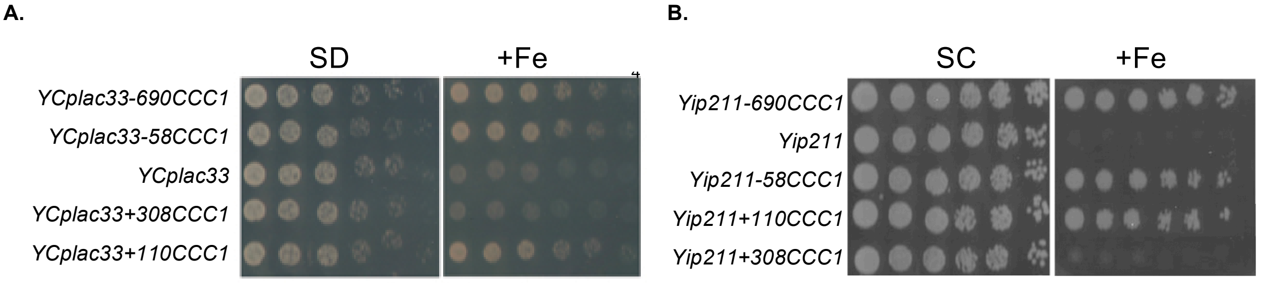

Figure S2. Removal of *CCC1* promoter and part of the gene coding sequence, still rescues Δ*ccc1* high iron–associated phenotype, independently of the vector used for cloning. The fragments *-690CCC1*, *-58CCC1*, *+110CCC1*, *+308CCC1* were cloned into (A) the centromeric vector YCplac33 or (B) the integrative vector YIplac211. The resulting constructs were used to transform a Δ*ccc1* strain. Exponentially growing cells were serially diluted and spotted onto control SC/SD plates or SC/SD plates supplemented with $FeSO_4$ (+Fe).

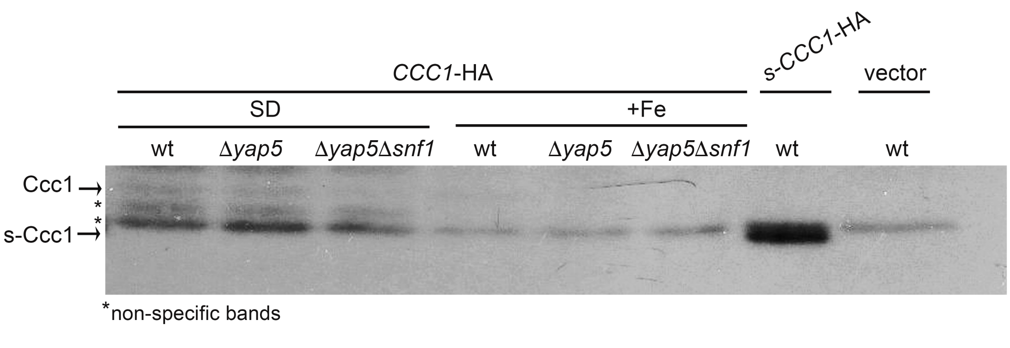

Figure S3. Deletion of *YAP5* and *SNF1* does not induce s-Ccc1 expression. Wild type, *Δyap5 and Δyap5Δsnf1* strains were transformed with either *CCC1*-HA (*-690CCC1-HA*), *s-CCC1*-HA (*+110CCC1-HA)* or the empty vector (pRS416) and left untreated (SD) or treated (+Fe) with 5mM $FeSO_4$ for 4h. Protein extracts were immunoblotted with an anti-HA antibody.

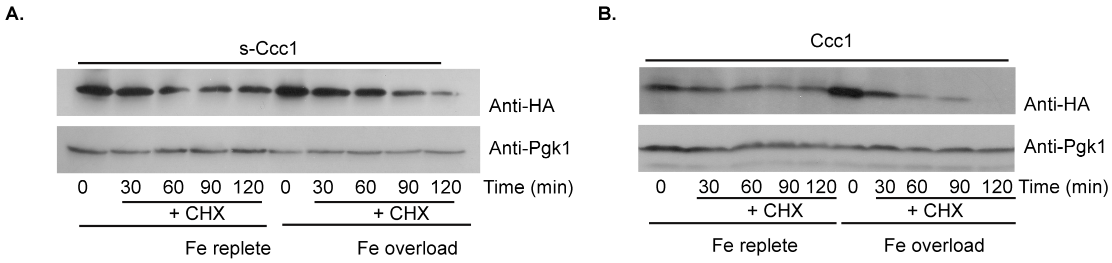

Figure S4. s-Ccc1 and Ccc1 stability. Ccc1-HA and s-Ccc1-HA protein stability was assessed by cycloheximide (CHX) chase assay. Δ*ccc1* expressing either the s-Ccc1 (A) or Ccc1 (B) (plasmids *+110CCC1-HA* and *-690CCC1-HA*, respectively) were grown to mid-exponential phase and left untreated or treated with 2 mM of $FeSO_4$ for 30 minutes. CHX was then added to a final concentration of 100 µg/mL. Cells were collected at the indicated time points and protein extracts were immunobloted using an anti-HA antibody. Pgk1 levels were used as a loading control.

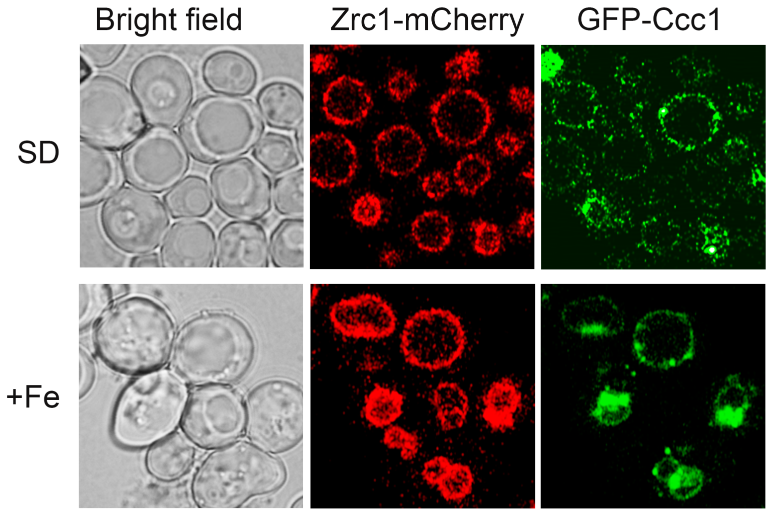

Figure S5. Ccc1 is located in the vacuole membranes. A strain with a genomic mCherry tagged copy of *ZRC1* was transformed with a plasmid encoding the fusion GFP- Ccc1. Cells were grown to mid-exponential phase and collected. Live cells were imaged by confocal microscopy under control conditions (SD) and iron overload conditions (+Fe).

Table S1. *Saccharomyces cerevisiae* strains used in this study.

Strain	Genotype	Source
BY4742	MATα, *his3Δ1 leu2Δ0 met15Δ0 ura3Δ0*	EUROSCARF
Δ*ccc1*	MATα, *his3Δ1 leu2Δ0 met15Δ0 ura3Δ0 YLR220w::kanMX4*	EUROSCARF
ZRC1-mCherry	MATα, *his3Δ1 leu2Δ0 met15Δ0 ura3Δ0 YMR243c-mCherry his3*$^+$	This study
Ylp2111-690CCC1	MATα, *his3Δ1 leu2Δ0 met15Δ0 ura3Δ0 YLR220w::kanMX4 URA3::-690CCC1 ura3*$^+$	
Ylp2111-58CCC1	MATα, *his3Δ1 leu2Δ0 met15Δ0 ura3Δ0 YLR220w::kanMX4 URA3::-58CCC1 ura3*$^+$	This study
Ylp2111+110CCC1	MATα, *his3Δ1 leu2Δ0 met15Δ0 ura3Δ0 YLR220w::kanMX4 URA3::+110CCC1 ura3*$^+$	This study
Ylp2111+308CCC1	MATα, *his3Δ1 leu2Δ0 met15Δ0 ura3Δ0 YLR220w::kanMX4 URA3::+308CCC1 ura3*$^+$	This study
Δ*yap5*	MATα, *his3Δ1 leu2Δ0 met15Δ0 ura3Δ0 YIR018w::kanMX4*	EUROSCARF
Δ*snf1*	MATα, *his3Δ1 leu2Δ0 met15Δ0 ura3Δ0 YDR477w::his3MX6*	This study
Δ*yap5*Δ*snf1*	MATα, *his3Δ1 leu2Δ0 met15Δ0 ura3Δ0 YIR018w::kanMX4 YDR477w::his3MX6*	This study

Table S2. Oligonucleotides used in the study.

Name	Sequence 5'- 3'	Use
CCC1_A1	TACAGGACCAAACCCCTCAG	*-690CCC1*
CCC1_A4	GGTTATCATGGATCACCTGA	Several *CCC1* constructs
pCCC1-240	CTTTGTTAGCCCGAATGTCTC	*-240CCC1*
pCCC1-198	GATTATATATGAAAAATTGATACAAAAC	*-198CCC1*
pCCC1-123	GTTAAAGCGACATCACTCTC	*-123CCC1*
pCCC1-114	GGTTTATAGAATAGAAATATAAC	*-114 CCC1*
pCCC1-90	GAACGAAGCCTTTAGACTCT	*-90CCC1* and *-90CCC1-HA*
pCCC1-58	TGGTATATTGAACAAAGAAA	*-58CCC1, -58CCC1*-HA and yEp356R-58
qCcc1_Fw	AACGCAGTGGTGACCCTTAT	qRT-PCR
qCcc1_Rev	TCCTGCGCTGAATTTCTACC	
CCC1_DM_HA_Fw	CTTTGTTAAGTTACTGGGTATGTACCCATACGATGTTCCAGATTACGCTTAAG TGTAAAGTAACAACAC	-690*CCC1-HA*
CCC1_DM_HA_Rv	GTGTTGTTACTTTACACTTAAGCGTAATCTGGAACATCGTATGGGTACATAC CCAGTAACTTAACAAAG	
EcoRI_pCCC1_Rv	ggGAATTCCCTCAACAAGGGAGTTGAT	yEp356R-58
IN_ORF_CCC1	ATGTCCATTGTAGCACTAAAGAA	yEp356R+1
IN_ORF2_CCC1	GTAGTAATAGCAATAGTTCAAG	*+110CCC1*, *+110CCC1-HA*
IN_ORF3_ CCC1	ATGTGATTATCGGGCTAAGC	*+308CCC1*
mATG_leu_2_Fw	AACTCACCGtTGTCAGTAGGG	*+110CCC1*^{mut}*-HA*
mATG_leu_2_Rv	CCCTACTGACAaCGGTGAGTT	
Frame_2_Fw	AATATTATGTCgCATTGTAGCACT	*-690CCC1*^{frame}*-HA* and *-90CCC1*^{frame}*-HA*
Frame_2_Rv	AGTGCTACAATGcGACATAATATT	
BamHI_mCherry_Fw2	ggCGGATCCCCGGGTTAATTAACAGTATGGTCAGCAAGGGAGAGGAAGATA A	pFA6a-mCherry-His3MX6
AscI_mCherry_2_Rv	ggcgcgccCTATTTGTATAATTCGTCCAT	
Zrc1_F2	gctgtaaactgcaatacttccaattgcctgCGGATCCCCGGGTTAATTAA	ZRC1 C-terminal mCherry tag K7
Zrc1_R1	agtatatagtttatgttctctgtagaaccatGAATTCGAGCTCGTTTAAAC	
CCC1_2ATG_Fw	ATGTCAGTAGGGAAAGATAATAGGA	GFP-s-*CCC1*
HA_ClaI_rv	ccacatcgatAGCGTAATCTGGAACATC	GFP-s-*CCC1*
T7_CCC1_Rv	TAATACGACTCACTATAGG CCAGTAACTTAACAAAGAACC	Northern blot
CCC1_F	AACGCAGTGGTGACCCTTAT	Northern blot probe
CCC1_R	ACCACCCACAACAACCATTT	Northern blot probe
Frame_2_Fw	AATATTATGTCgCATTGTAGCACT	*-690CCC1*^{frame}*-HA* and *-90CCC1*^{frame}*-HA*
Frame_2_Rv	AGTGCTACAATGcGACATAATATT	
pRS416_lacZ_Rv	GGGCTGCAGGAATTCGATATC	*-25CCC1-HA, +40CCC1-HA, +60CCC1-HA and +80CCC1-HA*
pRS416-25Fw	GAATTCCTGCAGCCC TCCCATATCTCGTGCACACAAATAT	*-25CCC1-HA*
pRS416+40Fw	GAATTCCTGCAGCCCTACAGAAAGCGAAAGGTAGTGGT	*+40CCC1-HA*
pRS416+60Fw	GAATTCCTGCAGCCCTGGTGGAACCTCAGAGTTGG	*+60CCC1-HA*
pRS416+80Fw	GAATTCCTGCAGCCCGGGGGTCTGAATCAACTCCC	*+80CCC1-HA*
SNF1_HIS_Fw	TTTGTAACAAGTTTTGCTACACTCCCTTAATAAAGTCAAC ATCGTACGCTGCAGG	Disruption of *SNF1*
SNF1_HIS_Rv	AACTTCCATATCATTCTTTTACGTTCCACCATCAATTGC TTAGGGAGACCGGCAGAT	

Table S3. Plasmids used and generated in this study.

Plasmid Name	Description	Reference
-690CCC1	PCR amplification from genomic DNA (BY4742, WT) using CCC1-A1 and CCC1-A4 primers and cloning into pRS416 (SmaI)	[1]
**pRS416**	*CEN, URA*	[2]
-273CCC1	*-690CCC1* digested with KpnI and cloned into pRS416 (KpnI, SmaI)	[1]
-240CCC1	PCR amplification of *-690CCC1* using CCC1-240 and -A4 primers and cloning into pRS416 (SmaI)	This study
-198CCC1	PCR amplification of *-690CCC1* using CCC1-198 and -A4 primers and cloning into pRS416 (SmaI)	This study
-114CCC1	PCR amplification of *-690CCC1* using CCC1-114 and -A4 primers and cloning into pRS416 (SmaI)	This study
-90CCC1	PCR amplification of *-690CCC1* using CCC1-90 and -A4 primers and cloning into pRS416 (SmaI)	This study
-58CCC1	PCR amplification of *-690CCC1* using CCC1-58 and -A4 primers and cloning into pRS416 (SmaI)	This study
-690CCC1-HA	*-690CCC1* with a HA epitope before the stop codon. *CCC1* was amplified using a three-step-PCR strategy as described previously [1] and cloned into pRS416 (SmaI)	This study
-90CCC1-HA	PCR amplification of *-690CCC1-HA* using CCC1-90 and CCC1-A4 primers and cloning into pRS416 (SmaI)	This study
-58CCC1-HA	PCR amplification of *-690CCC1-HA* using CCC1-58 and CCC1-A4 primers and cloning into pRS416 (SmaI)	This study
**YEp356R-58**	PCR amplification of *-690CCC1* using CCC1-58 and EcoRI_pCCC1_Rv and cloning in yEp356R (SmaI, EcoRI)	This study
**YEp356R+1**	PCR amplification of *-690CCC1* using IN_ORF_CCC1 and EcoRI_pCCC1_Rv and cloning in yEp356R (SmaI, EcoRI)	This study
**YEp356R**	*2μ, URA, lacZ*	[3]
**YIp211-690CCC1**	PCR amplification of *-690CCC1* using CCC1-A1 and -A4 primers and cloning into YIplac211 (SmaI)	
**YIp211-*58CCC1***	PCR amplification of *-690CCC1* using CCC1-58 and -A4 primers and cloning into YIplac211 (SmaI)	This study
**YIp211+*110CCC1***	PCR amplification of *-690CCC1* using IN_ORF2_CCC1 and -A4 primers and cloning into YIplac211 (SmaI)	This study
**YIp211+*308CCC1***	PCR amplification of *-690CCC1* using IN_ORF3_ CCC1and -A4 primers and cloning into YIplac211 (SmaI)	This study
+110CCC1	PCR amplification of *-690CCC1* using IN_ORF2_CCC1 and -A4 primers and cloning into pRS416 (SmaI)	
+308CCC1	PCR amplification of *-690CCC1* using IN_ORF3_ CCC1and -A4 primers and cloning into pRS416 (SmaI)	
+110CCC1-HA	PCR amplification of *-690CCC1-HA* using IN_ORF2_CCC1 and -A4 primers and cloning into pRS416 (SmaI)	This study
+110CCC1^{mut}-HA	PCR site directed mutagenesis of *+110CCC1-HA* ATG using mATG_leu_2_Fw and mATG_leu_2_Rv	This study
-690CCC1^{frame}-HA	PCR site directed mutagenesis of *-690CCC1-HA* using Frame_2_Fw and Frame_2_Rv	This study
-90CCC1^{frame}-HA	PCR site directed mutagenesis of *-90CCC1-HA* using Frame_2_Fw and Frame_2_Rv	This study
-25CCC1-HA	PCR amplification of *-90CCC1-HA* using pRS416-25Fw and pRS416_lacZ_Rv followed by *in vivo* bacterial assembly	This study
+40CCC1-HA	PCR amplification of *-90CCC1-HA* using pRS416+40Fw and pRS416_lacZ_Rv followed by *in vivo* bacterial assembly	This study
+60CCC1-HA	PCR amplification of *-90CCC1-HA* using pRS416+60Fw and pRS416_lacZ_Rv followed by *in vivo* bacterial assembly	This study
+80CCC1-HA	PCR amplification of *-90CCC1-HA* using pRS416+80Fw and pRS416_lacZ_Rv followed by *in vivo* bacterial assembly	This study
**pFA6a-mCherry-His3MX6**	PCR amplified Cherry with BamHI_mCherry_Fw_2 and AscI_mCherry_2_Rv cloned in pFA6a-GFP(S65T)His3MX6 (AscI, BamHI)	This study
**pFA6a-GFP(S65T)-His3MX6**	*HIS3*, GFP	[4]
**pUG36**	*CEN, URA, pMET17, GFP-*	GenBank: AF298791.1
**GFP-s-*CCC1***	PCR amplification of *CCC1-HA* using CCC1_2ATG_Fw and HA_ClaI_Rv primers and cloning into pUG36 (SmaI, ClaI), The plasmid is a fusion between GFP and +207 bp of *CCC1*.	This study
**GFP-*CCC1***	PCR amplification of *CCC1-HA* using IN_ORF_1_CCC1 and The plasmid is a fusion between GFP and +1 bp of *CCC1* HA_ClaI_Rv primers and cloning into pUG36 (SmaI, ClaI)	This study
***-261CCC1*-HA**	PCR amplification of *-690CCC1*-HA using CCC1-261 and -A4 primers and cloning into pRS416 (SmaI)	This study
***-240CCC1*-HA**	PCR amplification of *-690CCC1*-HA using CCC1-240 and -A4 primers and cloning into pRS416 (SmaI)	This study
***-198CCC1*-HA**	PCR amplification of *-690CCC1*-HA using CCC1-198 and -A4 primers and cloning into pRS416 (SmaI)	This study
***-123CCC1*-HA**	PCR amplification of *-690CCC1*-HA using CCC1-123 and -A4 primers and cloning into pRS416 (SmaI)	This study

Table S4. Putative transcription factors binding sites located in the *CCC1* gene region spanning 273 bp upstream the AUG codon, according to YEASTRACT (http://www.yeastract.com).

Transcription Factor	Consensus[a]	Position[b]
Msn2, Msn4, Nrg1, Rph1	CCCCT	-270
Rtg1, Rtg3	GGTAC	-268
Gis1, Msn2, Msn4, Rph1, Com2, Usv1	AGGGG	-265
Swi4	CACGAAA	-259
Rtg1, Rtg3	GTCAC	-257
Fkh1, Fkh2	AAACA	-242
Tec1	GAATGT	-229
Stb5	CGGNS	-228
Rox1	ACAAT	-220
Pho2	WTAWTW	-199
Mot2	ATATA	-191,-189
Mot2	ATATA	-189
Pho2	WTAWTW	-155
Ash1	YTGAT	-151
Pho2	WTAWTW	-94
Fkh1,Fkh2p	RYMAAYA	-56
Mot2	ATATA	-52

[a]N- any base pair, S- C or G, W- A or T, Y- C or T

[b]bp upstream the ATG triplet encoding the first Ccc1 methionine

References

1. Pimentel, C., et al., *The role of the Yap5 transcription factor in remodeling gene expression in response to Fe bioavailability.* PLoS One, 2012. **7**(5): p. e37434.
2. Sikorski, R.S. and P. Hieter, *A system of shuttle vectors and yeast host strains designed for efficient manipulation of DNA in Saccharomyces cerevisiae.* Genetics, 1989. **122**(1): p. 19-27.
3. Myers, A.M., et al., *Yeast shuttle and integrative vectors with multiple cloning sites suitable for construction of lacZ fusions.* Gene, 1986. **45**(3): p. 299-310.
4. Longtine, M.S., et al., *Additional modules for versatile and economical PCR-based gene deletion and modification in Saccharomyces cerevisiae.* Yeast, 1998. **14**(10): p. 953-61.
